# The use of mobile phone data for the estimation of the travel patterns and imported *Plasmodium falciparum *rates among Zanzibar residents

**DOI:** 10.1186/1475-2875-8-287

**Published:** 2009-12-10

**Authors:** Andrew J Tatem, Youliang Qiu, David L Smith, Oliver Sabot, Abdullah S Ali, Bruno Moonen

**Affiliations:** 1 Department of Geography, 3141 Turlington Hall, University of Florida, Gainesville, Florida, 32611-7315, USA; 2 Emerging Pathogens Institute, University of Florida, Gainesville, Florida, 32610-0009, USA; 3 Department of Biology, Bartram-Carr Hall, University of Florida, Gainesville, Florida, 32611, USA; 4 The William J Clinton Foundation, 383 Dorchester Avenue, Suite 400, Boston, Massachusetts, 02127, USA; 5 Zanzibar Malaria Control Programme (ZMCP), Zanzibar Ministry of Health and Social Welfare, P. O. Box 236, Zanzibar

## Abstract

**Background:**

Malaria endemicity in Zanzibar has reached historically low levels, and the epidemiology of malaria transmission is in transition. To capitalize on these gains, Zanzibar has commissioned a feasibility assessment to help inform on whether to move to an elimination campaign. Declining local transmission has refocused attention on imported malaria. Recent studies have shown that anonimized mobile phone records provide a valuable data source for characterizing human movements without compromizing the privacy of phone users. Such movement data in combination with spatial data on *P. falciparum *endemicity provide a way of characterizing the patterns of parasite carrier movements and the rates of malaria importation, which have been used as part of the malaria elimination feasibility assessment for the islands of Zanzibar.

**Data and Methods:**

Records encompassing three months of complete mobile phone usage for the period October-December 2008 were obtained from the Zanzibar Telecom (Zantel) mobile phone network company, the principal provider on the islands of Zanzibar. The data included the dates of all phone usage by 770,369 individual anonymous users. Each individual call and message was spatially referenced to one of six areas: Zanzibar and five mainland Tanzania regions. Information on the numbers of Zanzibar residents travelling to the mainland, locations visited and lengths of stay were extracted. Spatial and temporal data on *P. falciparum *transmission intensity and seasonality enabled linkage of this information to endemicity exposure and, motivated by malaria transmission models, estimates of the expected patterns of parasite importation to be made.

**Results:**

Over the three month period studied, 88% of users made calls that were routed only through masts on Zanzibar, suggesting that no long distance travel was undertaken by this group. Of those who made calls routed through mainland masts the vast majority of trips were estimated to be of less than five days in length, and to the Dar Es Salaam Zantel-defined region. Though this region covered a wide range of transmission intensities, data on total infection numbers in Zanzibar combined with mathematical models enabled informed estimation of transmission exposure and imported infection numbers. These showed that the majority of trips made posed a relatively low risk for parasite importation, but risk groups visiting higher transmission regions for extended periods of time could be identified.

**Conclusion:**

Anonymous mobile phone records provide valuable information on human movement patterns in areas that are typically data-sparse. Estimates of human movement patterns from Zanzibar to mainland Tanzania suggest that imported malaria risk from this group is heterogeneously distributed; a few people account for most of the risk for imported malaria. In combination with spatial data on malaria endemicity and transmission models, movement patterns derived from phone records can inform on the likely sources and rates of malaria importation. Such information is important for assessing the feasibility of malaria elimination and planning an elimination campaign.

## Background

Many countries are committing to nationwide malaria elimination and global eradication is once more back on the international agenda [1-3]. Historically, the technical feasibility of achieving malaria elimination in a region has been conceptualized as being composed of 'receptivity' and 'vulnerability' [4,5]. Receptivity represents the strength of transmission in an area, while vulnerability is the risk of malaria importation [6]. While both have been regularly discussed theoretically, neither have been quantified, nor methods for their quantification ever defined.

Quantifying imported malaria risk represents a central component for not only assessing the feasibility of malaria elimination from a region, but for planning the implementation of an elimination campaign. Malaria is constantly being exported and imported around the World, and in areas of high transmission, malaria importation is generally a minor concern. As local transmission is reduced and after malaria has been eliminated from a region, however, importation becomes a primary concern.

Zanzibar, an island group of the coast of Tanzania, is one of the territories in sub-Saharan Africa that has recently expressed its willingness to move from control towards elimination. Since 2003, the introduction of artemisinin-based combination therapy (ACT) and high coverages of long-lasting insecticide treated nets and indoor residual spraying, has reduced malaria prevalence to just 0.8% [7,8]. These efforts have resulted in the government of Zanzibar considering an elimination campaign and undertaking an elimination feasibility assessment. Nevertheless, proximity and high connectivity to the mainland where transmission levels remain substantially higher in many places [9] implies that imported malaria will be a constant problem [10].

In general, parasites can be imported into Zanzibar in one of three ways: (i) the migration of an infected mosquito, (ii) infected humans visiting or migrating from the mainland, (iii) residents visiting the mainland and becoming infected, then returning. While mosquitoes may occasionally arrive though wind-blown or accidental aircraft or ship transport, typically they will only fly short distances. Human carriage of parasites, therefore, represents the principal risk, and is to blame in many past instances elsewhere where malaria has resurged [11-14]. Quantifying such movements both temporally and spatially, and the resulting imported infection risks, represents an important task if effective, evidence-based planning for elimination is to be undertaken.

Recent approaches to quantifying human mobility patterns point the way to novel insights from new data [15,16], especially through the analysis of mobile phone records [17-19]. Anonimized phone call record data that has both the time each call was made and the location of the nearest mast that each call was routed through can be used to construct trajectories of the movements of individuals over time [19]. Here, the potential of such data for estimating importation risk in the malaria elimination feasibility assessment for the islands of Zanzibar is demonstrated. The low market share on the mainland for the network provider restricts the focus here to those infections brought in by residents returning from mainland travel. However, the approaches put forward are sufficiently generic to be applied to alternative regions, elimination settings and phone network provider data. Moreover, this exercise aims to present the first exploration of mobile phone based approaches to the quantification of vulnerability to inform malaria elimination decisions and planning.

## Methods

### Study area

**Figure 1:**
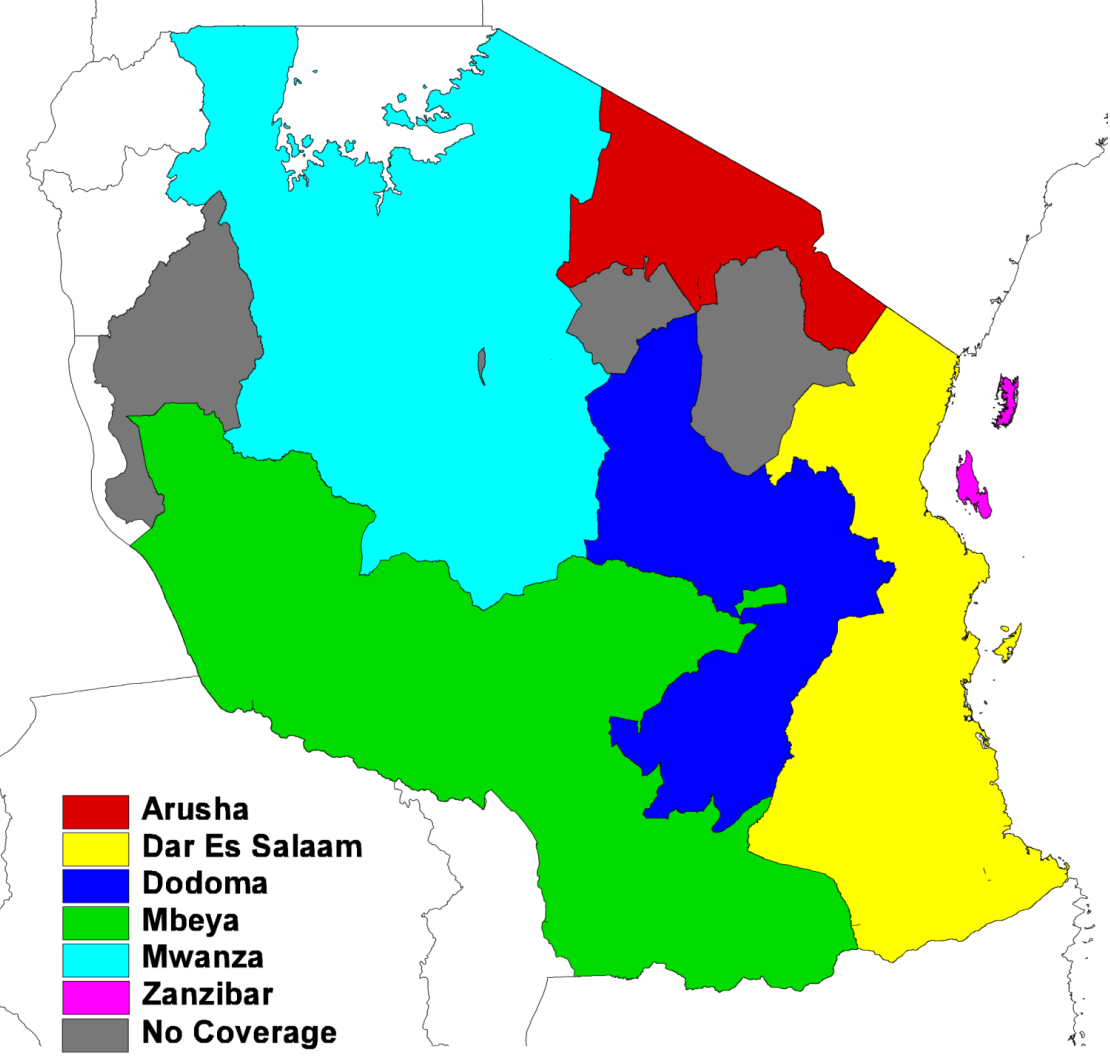
**Zantel coverage regions in Tanzania**.

Like other areas of sub-Saharan Africa, the islands of Zanzibar, off the coast of Tanzania in East Africa (Figure 1), have falciparum malaria and efficient vectors, including *Anopheles gambiae*, and at many points in the past, malaria in Zanzibar would have been called hyperendemic (*Pf*PR in the 2-10 age group ~50-75%). Recent control efforts [8], possibly combined with socioeconomic changes, have pushed *Plasmodium falciparum *prevalences down to 0.3% for the southern island of Unguja, and 1.4% for the northern island of Pemba [7], meaning approximately 8,500 infected people at any one time; 3,000 on Unguja and 5,500 on Pemba.

**Figure 2:**
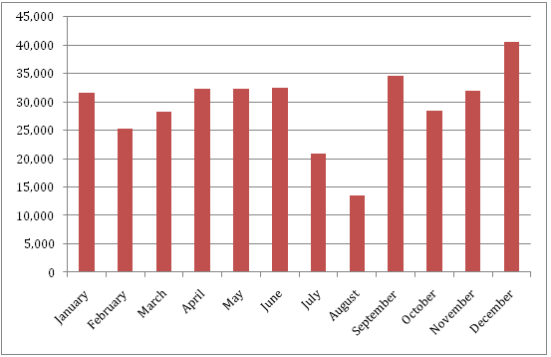
**Total ferry passenger numbers between Dar Es Salaam and Zanzibar for 2007**.

Zanzibar, however, has strong transport connections to the mainland where transmission levels are higher, resulting in concerns about achieving and sustaining elimination being raised [10], and making the quantification of human movement patterns and ultimately, imported infection rates, a critical aspect of elimination feasibility. While daily flights bring in around 10,000 people a month [20], these are mainly tourists from non-endemic regions, who will likely be taking prophylaxis, and thus represent a low risk in terms of imported infections and onward transmission. Ferry services have capacity to move up to 1,800 people daily between Zanzibar and the mainland. This route, as well as informal movements such as small fishing and trading vessels, likely represent the highest risk pathways for any imported infections. Figure 2 shows the recorded total numbers of ferry passengers each month for 2007, with these numbers likely split equally between visitors from the mainland and Zanzibar residents [21].

### *Plasmodium falciparum* malaria endemicity data

**Figure 3:**
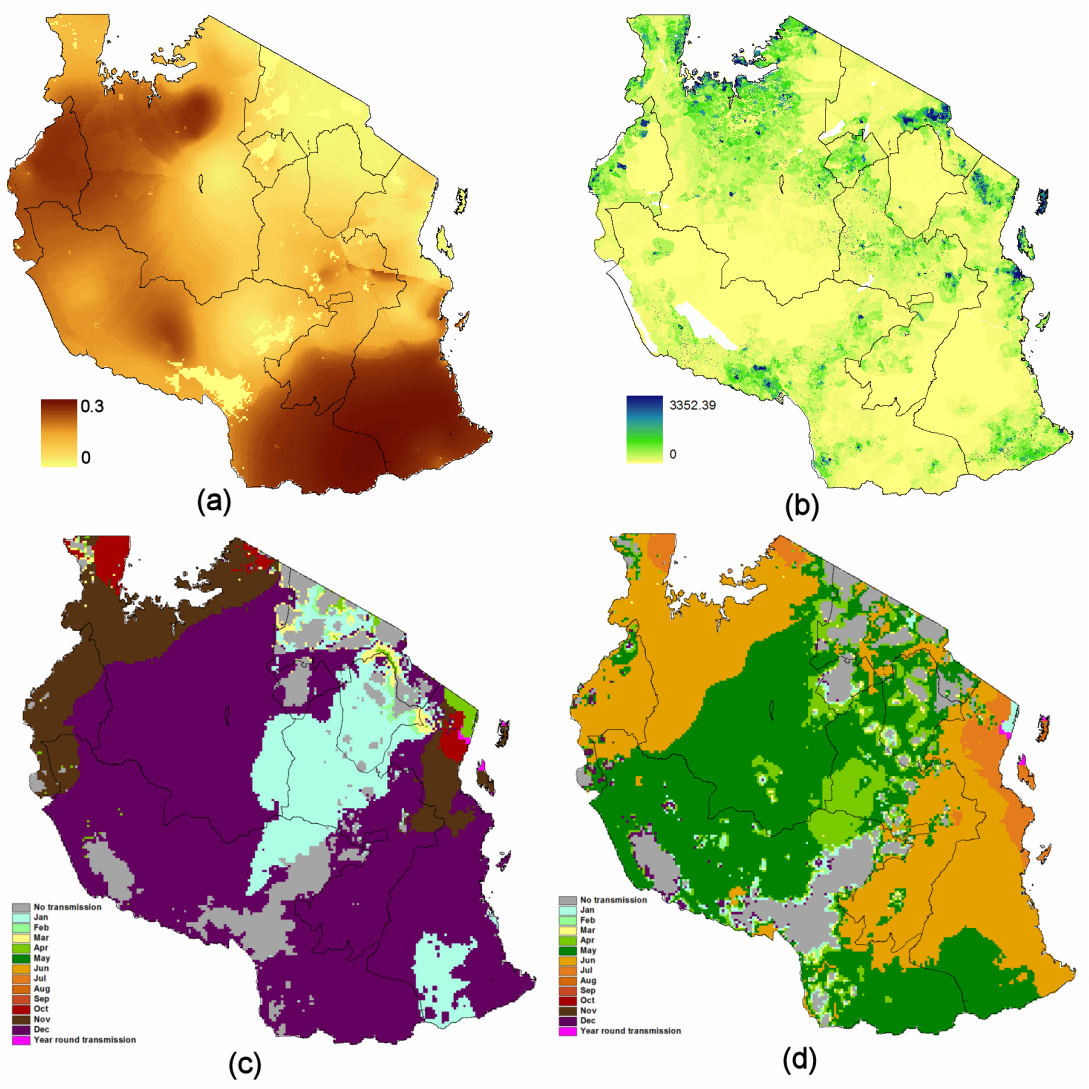
**Zantel coverage regions for Tanzania overlaid on (a) Daily Entomological Inoculation Rate (dEIR); (b) Population distribution; (c) Month of start of principal malaria transmission season; (d) Last month of principal malaria transmission season**. Secondary transmission season maps shown in Additional File 1: supplemental information.

A new global map of *P. falciparum *malaria endemicity for 2007 has now been published [9]. This provides a continuous prediction of prevalence (*P. falciparum *parasite rate in the two up to ten year old age group, PfPR2-10, between 0-100%) for every 5 × 5 km pixel within the stable limits of *P. falciparum *malaria transmission [22]. It represents a contemporary measure of global malaria endemicity, based on evidence in a huge repository of parasite rate surveys [23]. Using mathematical models described previously [24-26], the parasite rate map was converted to a map of the daily Entomological Inoculation Rate (dEIR), a more relevant measure for assessing the risk of infection acquisition in an area. The dEIR data for the study area was extracted and is shown in Figure 3(a). Maps showing the start and end months of the principal (Figures 3(c) and 3(d)) and secondary *P. falciparum *transmission seasons [27] extracted for Tanzania were also obtained to enable spatial refinement of transmission levels during the study period.

### Population distribution data

Population distribution maps for 2002 at 100 m spatial resolution, as described in Tatem *et al *[28] and available through the AfriPop project [29], were obtained for the study area. These were projected forward to 2008 to match the mobile phone data by applying national, medium variant, inter-censal growth rates [30] using methods described previously [31] and are shown in Figure 3(b).

### Mobile phone data

The Zanzibar Telecom (Zantel) mobile phone operator has approximately a 10% share of the Tanzanian market [32]. While nine out of ten Tanzanians are reported to have 'access' to a mobile phone, what these Figures mean in terms of ownership and usage are subject to debate and uncertainty [33,34]. However, while the 10% share Zantel has likely represents an unrepresentative sample of Tanzania as a whole, Zantel does have a 99% market share on Zanzibar. With over 330,000 individual users apparently resident on Zanzibar (see later analyses) out of a total population of just over a million, this suggests that a substantial sample of Zanzibar phone users is covered by the dataset. Analyses here were, therefore, focussed on Zanzibar residents only, though information derived from mainland resident users is presented in Additional file 1: supplemental information.

Records encompassing three months of complete mobile phone usage for the period October-December 2008 were obtained from Zantel. This represents the limit of available Zantel data, since the company only keeps the preceding three months of records. Nevertheless, this covers the busiest period in terms of travel to and from Zanzibar (Figure 2), and, therefore, enables a conservative upper limit on infection importation risk to be estimated. The data included the dates of all phone usage by 770,369 individual users, making a total of 21,053,198 calls and text messages. Prior to receiving the data, Zantel assigned each individual user a unique code to ensure that the anonymity of users was maintained and that the data could only be used for studying general patterns of mobility. Each individual call and message was spatially referenced to one of six areas: Arusha, Dar Es Salaam, Dodoma, Mbeya, Mwanza and Zanzibar (Figure 1). Any individual that made just four or less calls in any one month (an average of one per week) was removed from further analyses to ensure that sufficient temporal resolution existed in the remainder of the dataset for trajectory analysis.

### Estimating exposure to transmission levels

For each of the three months in the study period, and for each Zantel region, the areas within their principal (Figures 3(c) and 3(d)) or secondary transmission seasons were identified and overlaid onto the dEIR map (Figure 3(a)), with non-transmission season areas masked out. The minimum, mean and maximum dEIR values for each Zantel region and month were then calculated, and the gridded population data and dEIR data were combined to calculate population weighted mean dEIRs for the entire regions, and their principal cities (Table 1). To examine the ranges of possible results, should for instance the unlikely case of all visitors travelling to the highest transmission part of each Zantel region be reality, analyses were undertaken with the extreme conditions of minimum and maximum possible dEIR exposure per region. With Zantel coverage principally available in the major populated areas, and travellers more likely to visit heavily populated regions than empty rural areas, it was assumed however that the population-weighted measures likely represented the more realistic range of estimates for dEIR exposure within each coverage region. Moreover, with a high percentage of travellers likely visiting just the principal cities when travelling to each region, the population weighted mean dEIR within the city limits of Arusha, Dar Es Salaam, Dodoma, Mbeya and Mwanza, as defined by the global rural-urban mapping project urban extent map [35], were also calculated. These scenarios and assumptions were tested through comparing estimated imported infection numbers (see the quantifying imported malaria risk section) to the known numbers of infections present at any one time on the islands of Unguja and Pemba.

**Table 1:** Monthly estimates of dEIR for each Zantel region.

	Minimum			Mean			Maximum			Population weighted mean			Pop weighted principal city mean		
**Zantel region**	**Oct**	**Nov**	**Dec**	**Oct**	**Nov**	**Dec**	**Oct**	**Nov**	**Dec**	**Oct**	**Nov**	**Dec**	**Oct**	**Nov**	**Dec**

Arusha	0.0	0.0	0.0	0.00005	0.00021	0.00063	0.00068	0.00154	0.00341	0.00001	0.00008	0.00014	0	0.00006	0.00006
Dar Es Salaam	0.0	0.0	0.0	0.00104	0.00840	0.04049	0.00285	0.05654	0.21508	0.00082	0.00213	0.00855	0.00023	0.00023	0.00023
Dodoma	0.0	0.0	0.0	0.00032	0.00099	0.00792	0.00332	0.0322	0.05258	0.00021	0.00084	0.00785	0	0.00199	0.00199
Mbeya	0.0	0.0	0.0	0.00625	0.00974	0.02517	0.00995	0.06526	0.29468	0.00444	0.00848	0.03133	0	0.00512	0.00512
Mwanza	0.0	0.0	0.0	0.00031	0.00066	0.00846	0.00221	0.00887	0.03671	0.00009	0.00032	0.0169	0	0.00492	0.00492

### Quantifying imported malaria risk from returning residents

Malaria importation risk or vulnerability have been discussed in relation to malaria elimination for decades (e.g. [4,5,12]), but never quantified. In simple terms, malaria importation risk as a measurable quantity in a focal country or area is the product of human immigration rates from other malaria endemic countries or areas and their corresponding level of endemicity. However, it may not be sufficient to estimate the number of people who cross the borders of a country or region infected with malaria elsewhere; it also matters how long they stayed in endemic regions, how long they remain infected and infectious in the country or area of interest, as well as where they stay. Thus, the risks deriving from visitors from the mainland (see Additional file 1: supplemental information) and returning residents should be quantified differently.

For Zanzibar residents visiting the mainland on day *t*, their length of stay, *L*, and dEIR in the area of stay are the key factors. Recent research efforts have provided spatial quantification of *P. falciparum *endemicity [9] enabling estimation of the dEIR at the locations that Zanzibar residents are visiting. Motivated by malaria transmission models [36], the probability of obtaining an infection, *P*, is thus:

The total number of imported infections, *I*, over all *N *trips made in the three month study period is therefore:

Given the estimates of trip length, range of estimates of dEIR and proportion of travellers captured in the dataset, the total number of infections brought into Zanzibar by returning residents were estimated, as well as the distribution of infection origins. With only around 8,500 infections on the islands at any one time, and just 3,000 on Unguja, where the majority of movements to and from the mainland derive from, this places a realistic limit on the estimates of imported infection numbers, and thus, a guide to the likely dEIR visitor exposure for each Zantel region.

## Results

### Identifying travellers

Of the 770,369 individual phone users in the Zantel dataset, 24,625 (3.2%) made four calls or less per month in the three month study period, and were thus removed from further analysis. Of the remaining users, 335,621 made the majority of their calls on Zanzibar. From here on, we assume that these represent Zanzibar residents, since the majority of calls by a customer are most likely to be made in their home region. There will of course be exceptions to this, for instance, if a mobile phone is principally used for business use when travelling, but in the absence of further information, this represents a reasonable assumption to make. Of the 335,621 Zanzibar resident users, just 12.08% of them (40,543 users) made calls from the mainland. Thus, the vast majority of users only made calls from Zanzibar, indicating a lack of travel.

### Locations visited

**Figure 4:**
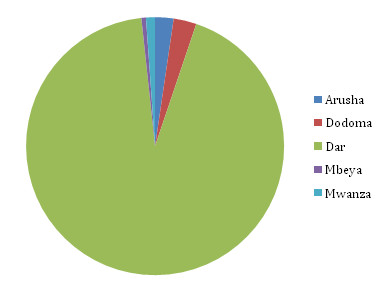
**The proportions of Zanzibar resident users that made the majority of their non-home calls at each location**.

Figure 4 shows, of those Zanzibar residents who travelled in the study period, the proportions that made the majority of their non-home calls at each other mast location. It is clear that of those who travelled to the mainland, a substantial proportion made the majority of their non-Zanzibar calls in the Dar Es Salaam region, with only a small proportion making the majority of their non-Zanzibar calls at the other four mast locations.

### Trip lengths

To estimate the lengths of trips made by those making calls from more than one location, it was assumed that the date of the first mainland call made represented the start of a trip. The end of this trip was estimated as the date when the first Zanzibar-based call was made again. For each user, the start and end dates of each individual trip made were estimated in this way and the trip lengths quantified and recorded. A total of 73,095 trips were made, with 12,584 residents travelling in October making a total of 24,439 trips, 11,947 in November making 24,335 trips and 12,882 in December making 24,321 trips. These figures correspond well with the ferry passenger numbers (Figure 1) and, assuming residents made up around half of ferry passengers [21], suggest that around 95% of all trips made by Zanzibar residents to the mainland were captured in the dataset.

**Figure 5:**
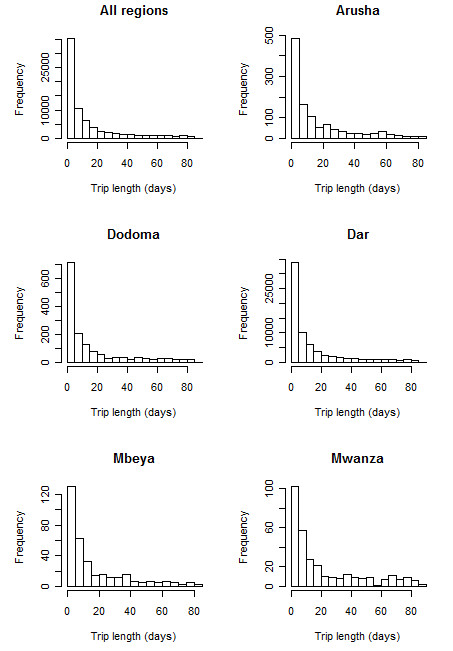
**The distributions of trip lengths made by Zanzibar residents to the mainland, overall and by Zantel region**. Note differing y-axis limits.

Figure 5 shows the distribution of trip lengths made by Zanzibar residents. As shown in Figure 4, the vast majority of trips made were to the Dar Es Salaam region. What is clear from Figure 5 is that the majority of trips made to the mainland were of less than five days long. In fact, 17.4% of all trips to the Dar region were estimated to be of just one day in length, while 29% were of two days in length or less. A similar pattern is shown for the other regions, though with substantially fewer visits made, and a higher proportion of longer (10-30 days) trips made by those travelling further, e.g. Mbeya or Mwanza.

### Estimating imported malaria risk

To provide estimates of imported case numbers from returning Zanzibar residents and likely origins of infections, the data on dEIR scenarios for each Zantel region were combined with the trip length estimates using equation (2). Table 2 shows that only the results from the population weighted region and city scenarios fall under the realistic limits of total infections on the islands, given that imported infections will also be brought in by visitors from the mainland and that the majority of travel is to Unguja. Realistically, while a significant majority of visitors to each region will visit the principal cities, others will travel to alternative population centres, thus the regional population weighted mean dEIR (upper) and principal city population weighted mean dEIR (lower) scenarios represent credible limits for estimating the likely number of imported infections per month arising from returning residents. Thus, converting these to annualized measures, estimates of between one and 12 imported infections per 1,000 people per year from returning residents represent realistic limits. Given increased travel in October-December (Figure 2), these also likely represent conservative overestimates.

**Table 2:** Estimated average monthly numbers of imported infections under the differing dEIR scenarios outlined in table 1.

Zantel region	Minimum	Mean	Maximum	Pop weighted mean	Pop weighted principal city mean
Arusha	0.0	1.84063	11.18779	0.47931	0.25034
Dar Es Salaam	0.0	3541.63122	8518.20389	1191.30602	81.11407
Dodoma	0.0	22.60743	127.97949	21.75056	10.77893
Mbeya	0.0	19.62360	53.46253	19.67336	6.03524
Mwanza	0.0	6.23628	22.12087	9.59984	6.88978

SUM	0.0	3591.93917	8732.95458	1242.80909	105.06836

**Figure 6:**
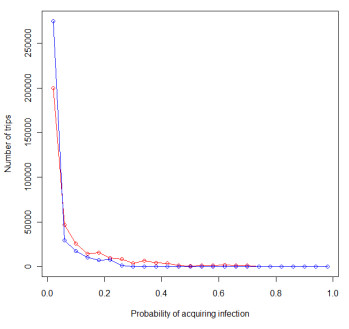
**All trips made by Zanzibar residents plotted by probability of infection acquisition, based on region population weighted mean dEIR (red line) and population weighted principal city mean dEIR (blue line)**.

**Figure 7:**
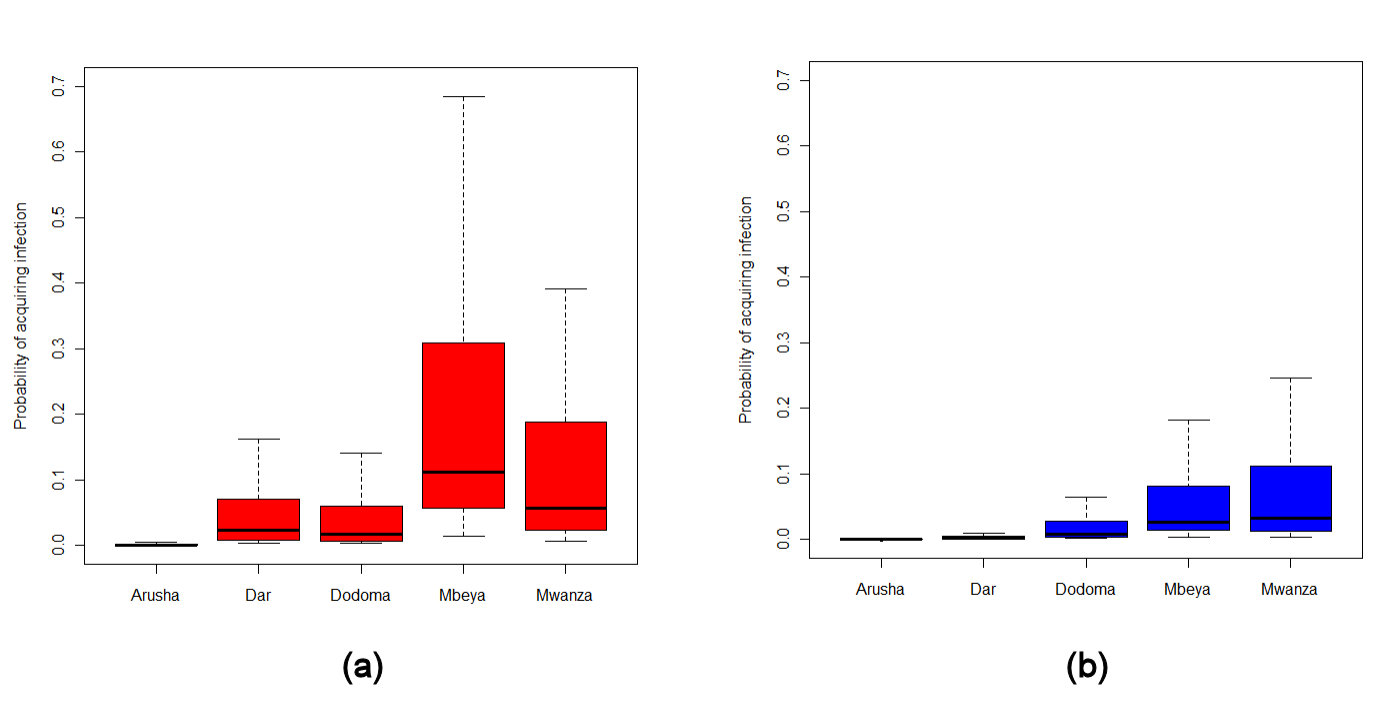
**Boxplots of trip probabilities of infection acquisition by Zantel region under scenarios of (a) region population weighted mean dEIR; (b) population weighted principal city mean dEIR**. The central dark line in each box shows the median value, the box size shows the interquartile range, while the whiskers extend to the most extreme datapoints that are no more than 1.5 times the interquartile range from the box.

Figure 6 shows the distribution of trips by probability of infection acquisition, *P*, under the scenarios of exposure to regional population weighted mean dEIR and principal city population weighted mean dEIR. Each scenario highlights that the majority of trips made entailed a probability of infection acquisition of less than 0.05. Figure 7 shows the regional composition of these distributions, illustrating that under both scenarios, the trips made by residents to Dodoma, Mbeya and Mwanza provided greater risks of infection acquisition, due to a higher proportion of longer stays in these regions typically, combined with overall high levels of transmission.

## Discussion

Results here show that, despite data limitations, spatially and temporally referenced mobile phone usage data can provide valuable information on human movement patterns. In combination with spatial data on malaria endemicity, derived movement patterns can inform on the likely sources, risks and case numbers of imported malaria. The estimates presented represent the first quantification of the vulnerability of an area to imported malaria, a necessary quantity in determining the feasibility of achieving and sustaining elimination.

According to the Zantel data, of the 770,369 users in the entire dataset (made up of Zanzibar and mainland residents), only just over 100,000 travelled anywhere during the three-month study period. Of those Zanzibar residents that travelled, the overwhelming majority went solely to the Dar Es Salaam region (and likely to Dar Es Salaam city itself), where the population weighted average dEIR is relatively low. The majority of these trips were for just one to two days, thus posing a relatively low risk of acquiring an infection and again confirming that most trips could not have involved travel to much further beyond Dar Es Salaam city itself. If malaria prevalence levels continue to fall on the nearby mainland [37,38], there is reason to believe that importation risk on Zanzibar will fall simultaneously. There do however exist small mobile groups that (i) travel for extended periods to the mainland from Zanzibar (ii) travel to higher transmission areas from Zanzibar. These represent the risk groups contributing most to the imported infection numbers brought in by residents visiting the mainland. Moreover, basic analyses on mainland resident movement patterns (Additional File 1: supplemental information), suggest that similar risk groups exist among visitors to Zanzibar.

As described in the methods section, the data used here have specific limitations that prevent more comprehensive analysis. With just a 10% share of the market on the mainland and Zantel subscribers more likely to travel to Zanzibar than non-subscribers, detailed analyses were not presented based on visitors from the mainland, since the data probably exhibits significant biases. In addition, the activities of visitors to high transmission areas are unknown - in extreme scenarios, some may sleep under bed nets in air-conditioned hotels, while others may spend the night outdoors. Further, those travelling to or from further afield than Tanzania are not captured by this dataset, nor are those who switch to an alternative network provider on the mainland, nor are trips longer than three months captured. Finally, information on movement patterns on Zanzibar are also lacking, preventing an understanding of the likelihood of onwards transmission, since imported cases may play a key role in sustaining local transmission in some parts of Zanzibar. Previous work has shown however, that many mobile phone companies often have the ability to provide more precise spatial locations on data (e.g[19]), potentially improving upon conclusions made, should similar malaria-related studies be undertaken. Moreover, additional studies are planned and should be encouraged to test the approaches presented here further and help to arrive at a clear methodology for the quantification of vulnerability. The importance of preserving the anonymity of phone users should remain the utmost priority though.

The information derived from these analyses can be used to guide strategic planning for elimination, should the Ministry of Health decide to pursue such a campaign. Typically, three principal means of reducing imported infection risk are considered: (i) Identify infected individuals and treat them promptly, ideally before or upon entry, before they can infect competent local vectors and lead to secondary cases and sustained foci of indigenous transmission; (ii) address the source of infection by directly reducing transmission in all regions that are primary sources of infected travellers; (iii) provide prophylaxis to residents visiting endemic areas. While the second method is being addressed indirectly through the scaling up of control on the mainland [37,38], these analyses provide baseline data to inform on the first and third approaches. Screening with rapid diagnostic tests (RDTs) or microscopy at the ports of entry and providing follow-up treatment of infected individuals may play an important role in reducing imported case numbers and outbreaks. Such an approach is being used for all individuals entering the island of Aneityum in Vanuatu [39], while visitors from Africa were tested at the airports of Oman during its elimination campaign. Moreover, the details of all visitors to Mauritius from endemic regions are recorded and follow-up is undertaken by health surveillance officers [40]. When movement rates are high and resources are limited however, as in the case of Zanzibar, screening all visitors at the ports or providing follow-up may be prohibitively expensive and inefficient due to the large number of low-risk trips undertaken (Figure 6).

Modelling work on achieving and maintaining elimination done for the Zanzibar malaria elimination feasibility assessment suggests that as long as effective coverage with vector control measures is higher than 80%, elimination will be achieved and can be maintained. However, once transmission is reduced to very low levels, scaling down prevention without risking resurgence will only be possible if the importation levels estimated here are lowered considerably [Moonen B, Cohen J, Smith DL, Tatem AJ, Sabot O, Msellem M, Le Menach A, Randell H, Bjorkman A, Ali A: Malaria elimination feasibility assessment in Zanzibar I: Technical feasibility. *Malar Journal *2009, in preparation]. Prophylaxis for Zanzibari travellers is unlikely to be cost-effective or even practical given the high frequency of travel to mainly low risk regions. Screening on the ferries, especially of high risk groups during high risk periods of the year, might be a simpler and more cost-effective option compared to screening at the port of entry. Passengers are on the slow and fast ferries for six and two hours, respectively; enough time to administer a short questionnaire a rapid diagnostic test and treatment if necessary. However, better data are necessary to determine the *Pf*PR in ferry travellers to appreciate the operational consequences of such an approach.

Future work will aim to link the findings here to GIS data on travel networks in the region, and build these into stochastic metapopulation models of transmission, providing flexible tools for elimination planning. Moreover, retrospective analyses of health facility records at Zanzibar malaria early epidemic detection system sites are being undertaken at present, while surveys on the ferries are planned to corroborate and compliment findings here. This work also links into and is complemented by other datasets being gathered and analysed as part of a new research agenda initiated by the Malaria Atlas Project [41] to quantify human movement patterns in relation to assessment of malaria elimination feasibility.

Malaria elimination requires a significant investment of resources and capacity and, as has been demonstrated twice before on Zanzibar, failure to achieve this ambitious target can lead to fatigue among donors and policymakers and subsequent devastating resurgence of malaria. As more countries across the world make progress toward malaria elimination, there is a need for evidence based and locally-tailored assessments of the feasibility of making the final step in initiating an elimination campaign. With mobile phone uptake continuing to grow around the world, this novel data source has the potential to play a key role in providing such valuable evidence. While 'vulnerability' has been discussed in relation to malaria elimination for decades, the approaches outlined here represent a first step towards finally quantifying it. Replicating and refining these approaches in other areas will enable the development of a standardized methodology for malaria importation risk assessment to aid countries that are considering and planning elimination.

## Conflicts of interests

The authors declare that they have no competing interests.

## Authors' contributions

AJT conceived, designed and implemented the research and wrote the paper. BM, DS, OS and YQ aided with ideas, methodological and editorial input. BM and AA provided support in data compilation. The final version of the manuscript was seen and approved by all authors.

## Supplementary Material

Additional file 1 **Supplemental Information**. Analyses of movement patterns of mainland residents based on mobile phone data. Click here for file
