# Supplementary material for: The use of mobile phone data for the estimation of the travel patterns and imported Plasmodium falciparum rates among Zanzibar residents
Source: Malar J. 2009 Dec 10;8:287. doi: 10.1186/1475-2875-8-287 (PMC2800118; doi:10.1186/1475-2875-8-287)
Supplement: Additional file 1 — Supplemental Information. Analyses of movement patterns of mainland residents based on mobile phone data. [file 1475-2875-8-287-S1.PDF]

<u>Supplemental materials: Analyses of movement patterns of mainland residents based on mobile phone data</u>

Location	No of users with majority of calls in this location	Percentage of users that were 'mobile'
Arusha	30403	14.55
Dodoma	26685	23.74
Dar Es Salaam	327394	13.43
Mbeya	17245	18.25
Mwanza	29917	10.58
Zanzibar	335621	12.08

Table 1. Numbers of users split by the location of the majority of their calls and the percentage of users at each location that made calls from more than one location

Table 1 presents counts of users by the mast for which the majority of their calls were routed through. From here on, we assume that these represent the home locations of these users, since the majority of calls by a customer are most likely to be made in their home region. There will of course be exceptions to this, for instance, if a mobile phone is principally used for business use when travelling, but in the absence of further information, we regard this as a reasonable assumption to make. Thus it can be seen from table 1 that the vast majority of customers using the Zantel network in Oct-Dec 2008 resided either on Zanzibar or in/near Dar Es Salaam.

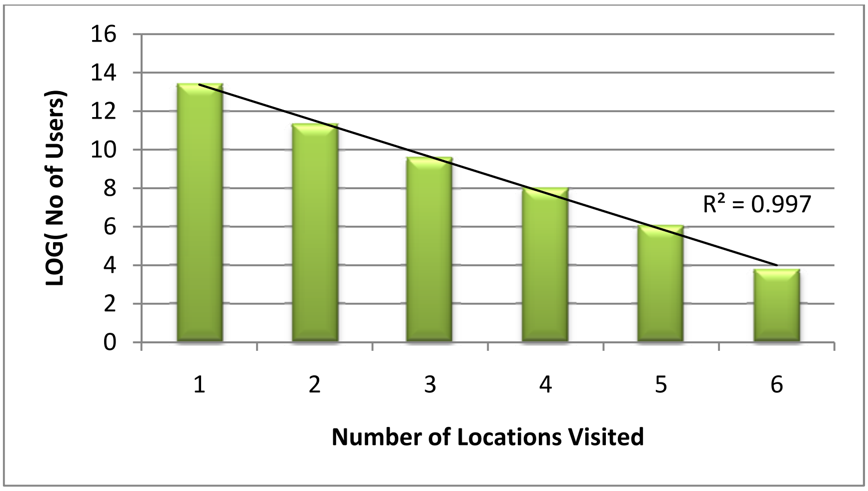

Figure 1. Number of locations visited against the log of number of customers, with linear trendline added.

Figure 1 demonstrates that mobility in the region follows the power law distribution that is found in other studies of human mobility (e.g. Brockmann et al 2006, Brockmann and Theis 2008, Gonzales et al 2008). This provides some measure of confidence that the dataset is a representative sample of human mobility in the region. It also outlines how mobile the entire set of users is over the 3 month period under study. It shows that the vast majority of users only make calls from one region (their 'home' region), with fewer and fewer making calls from 2,3,4,5 or all 6 regions.

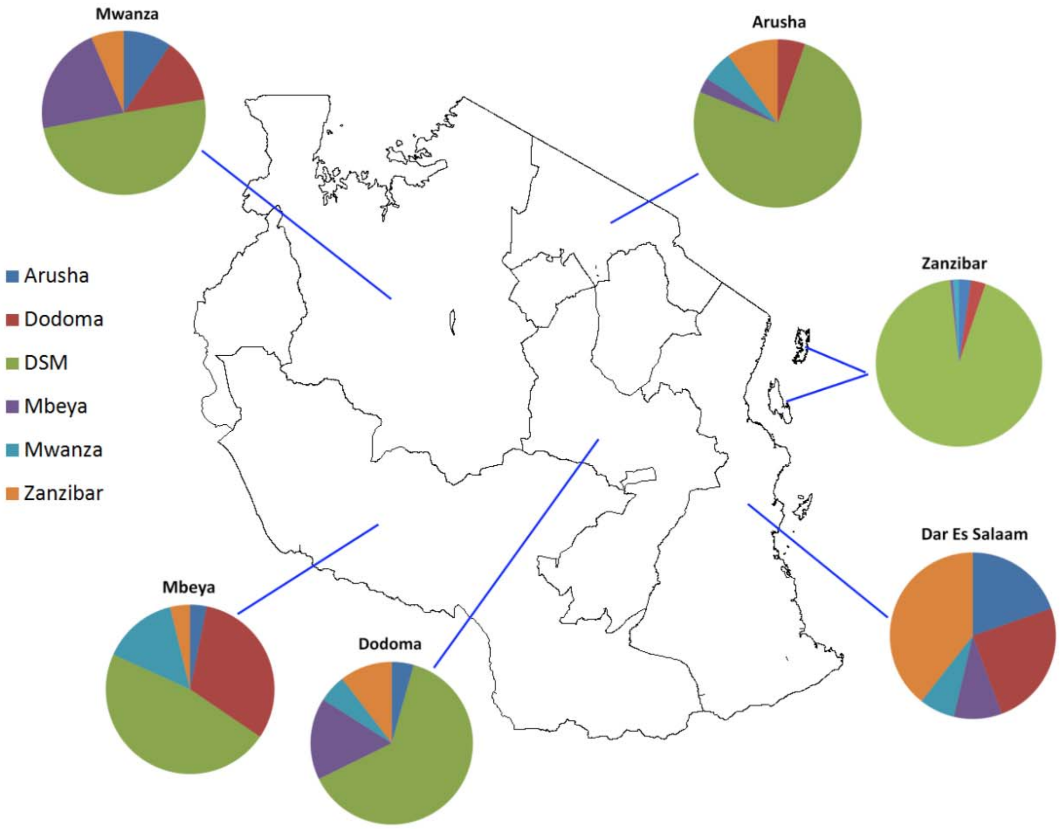

Figure 2. The proportions of Zanzibar resident users that made the majority of their non-home calls at each location.

Figure 2 shows, of the resident mobility user subgroups for each region, the proportions which made the majority of their non-home calls at each other mast location. It is clear, for instance, that of the mobile user subgroup on Zanzibar, the vast majority made the majority of their non-Zanzibar calls in Dar Es Salaam, with only a small proportion making the majority of their non-Zanzibar calls at the other four mast locations. Of those based elsewhere, the mobility user subgroup in Dar Es Salaam made the highest proportion of their non-home calls in Zanzibar (39%), with the proportions for Mwanza, Arusha, Dodoma and Mbeya being 6.5%, 10.1%, 10.5% and 3.8% respectively.

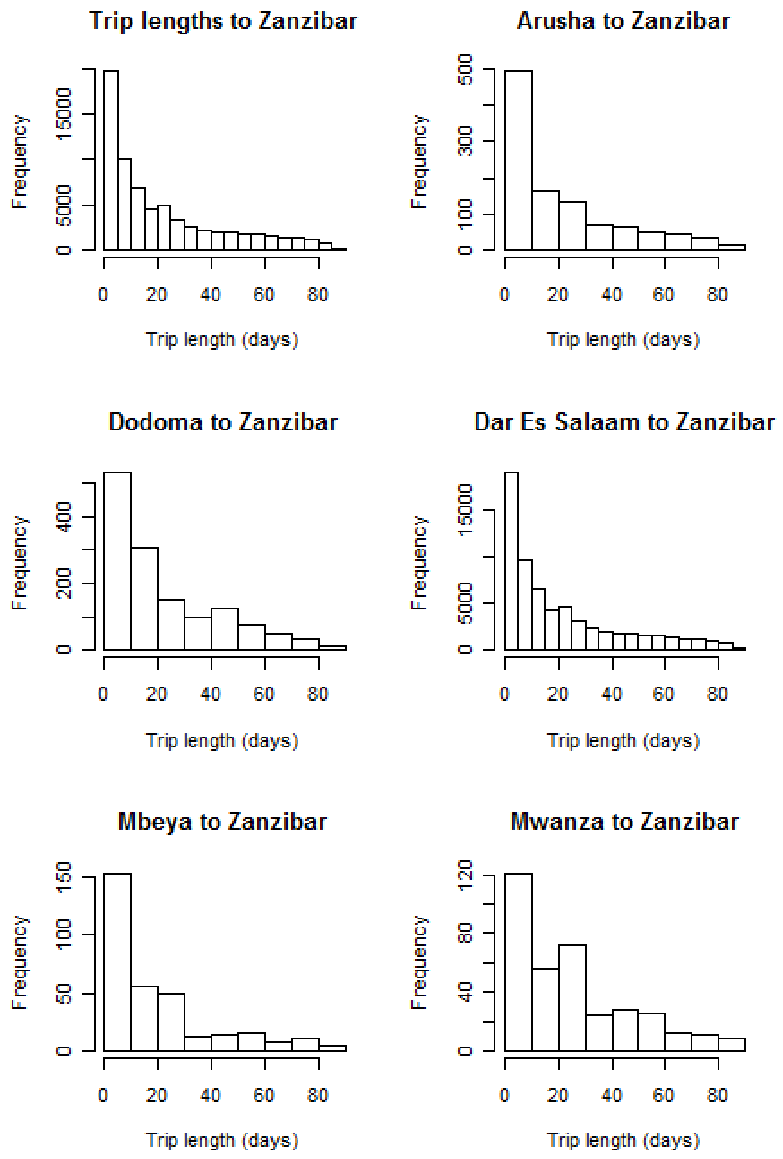

Figure 3. The distributions of trip lengths made by mainland residents to Zanzibar by Zantel region.

Figure 3 shows the distribution of trip lengths made by mainland residents to Zanzibar. The vast majority of trips made were from the Dar Es Salaam region and were of less than 5 days long. A similar pattern is shown for the other regions, though with substantially fewer visits made, and a higher proportion of longer (10-30 days) visits made by those travelling from further away, e.g. Mbeya and Mwanza.
